# Determining methylation status of methylguanine DNA methyl transferase (MGMT) from formalin-fixed, paraffin embedded tumor tissue

**DOI:** 10.1016/j.mex.2014.06.001

**Published:** 2014-06-12

**Authors:** Francine B. de Abreu, Torrey L. Gallagher, Emmeline Z. Liu, Gregory J. Tsongalis

**Affiliations:** Dartmouth Medical School and Dartmouth-Hitchcock Medical Center and The Audrey and Theodor Geisel School of Medicine at Dartmouth, Department of Pathology, 1 Medical Center Drive, Lebanon, NH 03756, United States

**Keywords:** MGMT, O-6-Methylguanine-DNA methyltransferase, Glioblastoma, Methylation, PCR

## Abstract

O-6-methylguanine-DNA methyltransferase (MGMT) has been associated with resistance to alkylating agent cancer therapy in Glioblastoma (GBM), the most common and aggressive primary brain tumor in adults. Lower expression or silencing of the MGMT protein by promoter methylation has been reported to improve survival in patients with GBM [1]. This protocol describes bisulfite conversion, methylation sensitive PCR amplification and data analysis/interpretation.

This protocol differs from published protocols in that it:

• Describes a detailed method to measure MGMT using DNA extracted from solid tumor tissue. We have optimized the DNA extraction by using FFPE tissue blocks that contain greater than 50% tumor tissue, when non-tumor tissue was also present. Performance of this assay is compromised when lower quantities of tumor cells are used as the methylation status of tumor cells is diluted out by methylation status of normal cells.
• The measurement of MGMT could be further (enhanced) optimized using a percentage of methylation ration cutoff of 2 as methylated.
• The machine specifications detailed here are specific to measuring MGMT from PPFE tumor tissue.

## Graphical abstract

**Figure:**
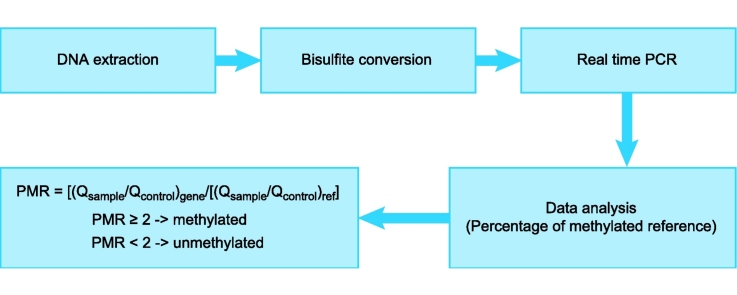

## Method details

### Specimen requirements

This assay is performed on tumor DNA isolated from FFPE tissue or fresh/frozen tissue. Quantity, purity and quality of extracted nucleic acids should be assessed by traditional methods before proceeding with this protocol. Appropriate block(s) or tissue specimens should be selected by the pathologist who reviewed the case. FFPE tissue blocks used should contain greater than 50% tumor tissue when non-tumor tissue is also present. If such a block is not available, tumor tissue can be dissected from an unstained section on a glass slide. Performance of this assay is compromised when lower quantities of tumor cells are used as the methylation status of tumor cells is diluted out by methylation status of normal cells.

*Note*: Sections fixed in a heavy metal fixative or specimens that have been decalcified are not acceptable for this procedure. Tissues fixed in heavy metal fixatives are known to inhibit downstream PCR amplification while decalcification of tissues is known to significantly degrade genomic DNA.

### Major equipment

• Bio-Rad DNA Engine^®^ Thermal Cycler (PTC-200)
  (Bio-Rad Laboratories)
• 7500 Fast Real-Time PCR System

(Applied Biosystems)

### Major reagents

• MethylEdgeTM Bisulfite Conversion System
  (Promega, Part # N1301)
• EpiTect MethyLight PCR + ROX Vial Kit
  (Qiagen, PN 59496)
• CpGenome Universal Methylated DNA
  (Millipore, PN S7821)
• CpGenome Universal Unmethylated DNA
  (Millipore, PN S7822)
• Converted Methylated Human Control DNA
  (Promega, PN N1221)
• Custom TaqMan^®^ Gene Expression Assay for MGMT (Assay ID AI89KYR) (Applied Biosystems, PN 4331348)
  MethyLight_MGMT_F: GCGTTTCGACGTTCGTAGGT
  MethyLight _MGMT_R: CAAACACTCACCAAATCGCAAA
  MethyLight _MGMT_Probe: TTCGCGGTGCGTATC
• Custom TaqMan^®^ Gene Expression Assay for MethyLight_ACTB (Assay ID AI5IQF3) (Applied Biosystems, 4331348)
  MethyLight_ACTB_F: TGATGGAGGAGGTTTAGTAAGTTTT
  MethyLight _ACTB_R: CACCACCCAACACACAATAACAA
  MethyLight _ACTB_Probe: TGGATTGTGAATTTGTG

### Quality control/process control

• Positive Control. Each run must contain two positive controls that consist of methylated DNA. The CpGenome Universal Methylated DNA (Millipore) has to be diluted to 10 ng/uL for optimal results and bisulfite converted. The Converted Methylated DNA (Promega) needs to be diluted to 3 ng/μL.
• Negative Control: Each run must contain one negative control that consists of unmethylated DNA. The CpGenome Universal Unmethylated DNA (Millipore) has to be diluted to 10 ng/μLfor optimal results and bisulfite converted.
• No Template Control (NTC). Each run must contain a no template control (NTC) that consists of all reagents with the exception of any DNA sample.

*Note*: The bisulfite conversion of the controls must be done at the same time as the samples.

### DNA conversion: MethylEdge Bisulfite Conversion System (Promega)[2]

*All reagents are stable at room temperature*

a. Prepare 1X ME Wash Buffer

• Add 24 mL of 95–100% ethanol to the bottle containing 6 mL of concentrated ME Wash Solution.

Mark on the bottle that you have performed this step.

b. Prepare Samples

• In a 0.2 mL PCR tubes, dilute 50 ng of each sample and control DNA in a total volume of 20 μL. Assay performance was optimized for 50 ng DNA because in small tissue biopsies large quantities of DNA may not be obtained.

*If the sample volume is less than 20 mcl, adjust the volume to 20 mcl with nuclease-free water*.

*You should prepare 3 tubes of the methylated DNA and 2 of the unmethylated DNA*.

c. Bisulfite Conversion

• Add 130 mcl of Bisulfite ME Conversion Reagent to each DNA sample and control, and pipet “up and down” to mix.
• Centrifuge briefly to collect the sample at the bottom of the tube.
  Ensure that the cap of the ME Conversion Reagent is closed tightly before storing the remaining reagent.
• Perform the conversion reaction using the cycling parameters shown in Table 1 on the Bio-Rad DNA Engine^®^ Thermal Cycler (Vf = 150 mcl).

According to the Manufacture's protocol, samples can be storage at 4 °C or on ice (protected from light) for up to 20 h.

d. DNA Desulphonation and Cleanup

• For each sample to be processed, place a ME Spin Column into one of the Collection Tubes.
• Add 600 mcl of ME Binding Buffer to the ME Spin Column.
• Transfer the entire bisulfite-treated sample to the column.
• Invert the tube several times (∼15×).
• Spin at maximum speed ≥10,000 × *g* for 30 s.
• Discard the flow through and re-insert the ME Spin Column into the same collection tube.

• Add 100 mcl of 1× ME Wash Buffer (with ethanol added).
• Spin at maximum ≥10,000 × *g* for 30 s.
• Add 200 mcl of ME Desulphonation Buffer to each ME Spin Column.
• Incubate at room temperature for 15 min.
• Spin at maximum ≥10,000 × *g* for 30 s.
• Add 200 mcl of ME Wash Buffer (ethanol added).
• Spin at maximum ≥10,000 × *g* for 30 s.
• Repeat this wash step once more.
• Place the ME Spin Column into a clean 1.5 mL microcentrifuge tube.
• Add 15 mcl of ME Elution Buffer.
• Incubate for 1 min at room temperature.
• Spin at maximum ≥10,000 × *g* for 30 s.
• Remove and discard the ME Spin Column.
• Store the DNA at −20 °C (protected from light).

*Pool the CpGenome Universal Methylated DNA that was converted (total of 3 tubes) into one tube*

*Pool the CpGenome Universal Unmethylated DNA that was converted (total of 2 tubes) into one tube*

### MethyLight reaction set-up [3]

*Thaw all reagents on ice, vortex and briefly centrifuge all reagents before dispensing*.

*Prepare 3 ng/mcl dilution of the Converted Methylated DNA (Promega) (dilution can be stored in the −20 °C freezer)*.

*One PCR reaction must be run for each sample to be tested*.

a. In 5 clean labeled 0.2 mL PCR tubes, make 5 serial dilutions 1:5 of the converted CpGenome Universal Methylated DNA as shown in Table 2.
  *CpGenome Universal Methylated DNA was converted in the previous step*.
b. In two clean labeled 1.5 mL microcentrifuge tube prepare the MGMT and ACTB master mixes as shown in Table 3.
c. Add 16 mcl of the MGMT Master Mix to the appropriate wells of a MicroAmp® Fast Optical 96-well Reaction Plate.
d. Add 16 mcl of the ACTB Master Mix to the appropriate wells of a MicroAmp^®^ Fast Optical 96-well Reaction Plate.
e. Add 4 mcl of standard control to the appropriate wells of the 96-well plate.
  *Mix the volume by pipetting up and down*.
f. Add 4 mcl of methylated control, unmethylated control, converted control and samples to the appropriate wells of the 96-well plate.
g. Seal the plate with a MicroAmp^®^ Optical Adhesive Film.
h. Briefly centrifuge the 96-well plate to ensure that all volume is collected in the bottom of the wells and that no bubbles are present.

### AB 7500 Fast Real-Time PCR System Set-up [4]

#### Experimental properties

• Turn on the ABI 7500 FAST real-time PCR system and the corresponding computer.
• Open the 7500 Fast System software program (7500 Software v2.0) by double clicking on the 7500 software icon located on the desk top.

*Alternatively, if the icon is not found you may load the system software by selecting “start”, “Programs”, “Applied Biosystems”, “7500 Software”, “7500 Software v2.0”*.

• Log into the Software package by selecting an existing user from the drop down menu or by clicking on “Login as guest”.
• In the home screen of the 7500 software, click on the “Advanced Setup” icon.

*Alternatively, select “Template” icon and load “MGMT Methylation Analysis” template proceed with Step 2 (Plate Set-up)*.

• If the “Advanced Setup” icon is not seen in the home screen, click on the arrow beneath the “Design Wizard” icon to expand the setup menu.
• In the Experiment Properties window under “How do you want to identify this experiment?” enter the run name, user name and any comment
• Under “Which instrument are you using to run the experiment?” select 7500 Fast (96 wells).
• Select “Quantitation – Standard curve” under the heading “Which type of Experiment do you want to set up?”.
• Under “Which reagents do you want to use to detect the target sequence?” select “TaqMan^®^ Reagents”.
• Under “Which ramp speed do you want to use in the instrument run?” select “Standard ∼2 h”

#### Plate set-up

• lick on the “Plate Setup” icon on the left hand side of the window
• In the “Define target and Samples” window load the “I Quant” assay from the database by selecting “Add Saved targets” highlighting the appropriate assay and clicking on “Add Selected targets”
• In the “Define Samples” window add the appropriate number of samples to the “Sample” window.
• Add the appropriate sample name and identifiers to the added samples.
• In the “Assign targets and Samples” window, click on the “Define and Set-up Standards” icon.
• Enter the appropriate Standard Curve Values as shown in Table 4 in the “Define Standard curve” window.

• In the “Assign Wells” window, select the wells which correspond to the standard curve placement on the 96-well plate.
• Click on “Apply”.
• In the “View Plate Layout” window highlight the wells that correspond to the sample placement as shown on the “7500 Setup” tab of the “MGMT Worksheet”.
• Select the “I Quant” assay from the “Assign target s to the Selected Wells” window.
• Assign the defined samples to the appropriate wells according to the “MGMT Worksheet” by highlighting the wells in which the sample will be placed and selecting the “Assign” check box next to the sample name.

#### Run method

• lick on the “Run Method” icon on the left hand side of the window.
• lick on “Open Run Method”.
• Select “45 cycles” and click on “OK”.
• Verify that the reaction volume is set to 20 mcl.
• Verify the thermal profile, as shown in Table 5, and edit if necessary.

• Click on the “Start Run” icon on the top right hand corner of the screen.
• Be sure to save the run as a unique file name.

#### Data analysis

*When the run has completed, the software should automatically open the “Analysis” window*.

• In the Analysis window with all samples selected, verify the efficiency of each standard curve for MGMT and ACTB. The value should be between 90 and 110%.
• Verify the threshold already established by the software. Make sure that all samples and controls have the same threshold.

*The threshold should be in the middle of the exponential curve*.

• Then click on the “Analysis” icon on the left hand side of the screen.
• Export the results to an excel file.

*Select “Export”, then “Browse”* → *“Desktop”* → *“MGMT” folder. After that, select “Start Export”. The excel file with the data will be exported to the Ion folder, and then you can close the window*.

• Save the excel file generated by the software. Copy the “t” and “Quantity mean” values of each sample from the “Analysis” file and paste them onto the “MGMT Worksheet”.
• Automatically the PMR (Percentage of Methylated Reference) will be calculated.

Check the values!

PMR = ((Qsample/Qmethy control)MGMT/(Qsample/Qmethy control)ACTB) * 100

PMR≥2 → methylated

PMR<2 → unmethylated

### Interpretation of results, reference values and acceptance criteria

#### Interpretation of controls

1. The positive controls (CpGenome Universal Methylated DNA and Converted Methylated Human Control DNA) should be correctly called methylated (PMR ≥ 2).
2. The negative control (CpGenome Universal Unmethylated DNA) should be correctly called unmethylated (PMR < 2).
3. No amplification should be observed in the NTC wells.

#### Interpretation of samples

1. A sample should be reported as methylated if the PMR ≥ 2 and if the controls have the expected profile. The PMR cutoff was established based on accuracy data form the clinical validation of this assay.
2. A sample should be reported as unmethylated if the PMR < 2 and if the controls have the expected profile.

*If the ACTB of a specific sample does not amplify, the sample must be repeated. It cannot be called unmethylated*.

## Figures and Tables

**Table 1:** Bisulfite conversion.

STAGE/STEP	HOLD STEPS		
Temperature	98	54	4
Time (mm:ss)	8:00	60:00	Forever

**Table 2:** Serial dilutions for ACTB and MGMT Standard Curves.

Standard	Starting material	Volume starting material (mcl)	Volume nuclease-free H_2_O (mcl)
12 ng standard	12 ng standard	4	–
2.4 ng standard	2.4 ng standard	4	16
0.48 ng standard	0.48 ng standard	4	16
0.096 ng standard	0.096 ng standard	4	16
0.0192 ng standard	0.0192 ng standard	4	16

**Table 3:** PCR master mix reaction set-up for MGMT and ACTB.

Master mix	Final concentration	Lot	1×	X reactions^a^
Nuclease-free water	–		3.5	
50× ROX Dye solution	1.25×		0.5	
10× Primer/Probe	1×		2.0	
2× EpiTect MethyLight Master Mix	1×		10.0	
Total volume			16.00	

^a^ Standard curves + total of samples + 2 Positive controls + 1 Negative control + 1 pipetting error.

**Table 4:** Define standard curve value for 7500 quantitation – standard curve run.

Standard curve property	Value to be entered
# of points	5
# of replicates	2
Starting quantity	12
Serial factor	1:5

**Table 5:** 7500 run.

STAGE/STEP	PRE-PCR	AMPLIFICATION	
	Holding stage	Cycling (45 Cycles)	
		Denature	Anneal/Extend
Temperature	95	95	60
Time (mm:ss)	10:00	00:15	01:00
Data collection	No	No	Yes

## References

[1] Uccella S. (2009). J. Clin. Pathol..

[2] Promega Technical Manual: MethylEdge™ Bisulfite Conversion System.

[3] Qiagen EpiTect^®^ MethyLight PCR Handbook.

[4] Applied Biosystems 7500 FAST Real Time PCR Instrument user's manual. https://tools.lifetechnologies.com/content/sfs/manuals/cms_050334.pdf.

